# Association of Metabolic Dysfunction-Associated Fatty Liver Disease with Cognitive Impairment and All-Cause Dementia: A Comprehensive Review

**DOI:** 10.5152/tjg.2024.23629

**Published:** 2024-02-01

**Authors:** Eda Kaya, Yusuf Yılmaz

**Affiliations:** 1 Division of Medicine, Department of Hepatology and Gastroenterology, Universitätsklinikum Knappschaftskrankenhaus Bochum, Ruhr University Bochum, Bochum, Germany; 2 Department of Gastroenterology, Recep Tayyip Erdogan University School of Medicine, Rize, Turkey

**Keywords:** Cognitive dysfunction, dementia, insulin resistance, liver fibrosis, metabolic dysfunction-associated fatty liver disease

## Abstract

Metabolic dysfunction-associated fatty liver disease (MAFLD) is a significant public health concern, affecting one-third of the global population and posing a risk for progressive liver disease. MAFLD is characterized by hepatic steatosis and impaired metabolic status, which not only impact the liver but also other systems of the human body, making it a multisystemic disorder. Emerging evidence suggests that MAFLD and its associated pathological pathways may contribute to cognitive impairment, potentially through neuroinflammation and neurodegeneration. Studies have detected cognitive impairment in patients with MAFLD using magnetic resonance imaging, which revealed decreased brain volume and cerebral perfusion, in addition to self-reported cognitive tests. While numerous studies have demonstrated an association between MAFLD and cognitive impairment, the relationship between MAFLD and all-cause dementia remains controversial. However, the shared pathological pathways between MAFLD and dementia, such as systemic inflammation, insulin resistance, gut dysbiosis, hyperammonemia, and vascular dysfunction, indicate the possibility of a common prevention strategy for both diseases. In this review, we provide a summary of the current evidence regarding the association between cognitive impairment, all-cause dementia, and MAFLD.

**Main Points**

- The accumulating evidence shows a significant relationship between metabolic dysfunction-associated fatty liver disease (MAFLD) and cognitive impairment.
- The relationship is possibly due to shared metabolic factors such as systemic inflammation, hyperammonemia, insulin resistance, gut dysbiosis, and vascular dysfunction.
- The association of MAFLD with all-cause dementia remains controversial.

## Introduction

Nonalcoholic fatty liver disease (NAFLD) is characterized by the presence of hepatic steatosis, diagnosed either through imaging or biopsy, after excluding secondary factors contributing to hepatic fat infiltration. It poses a significant public health concern, affecting approximately 30% of the global population and having the potential to progress to advanced liver disease.^1^ In 2020, a new definition, metabolic dysfunction-associated fatty liver disease (MAFLD), was proposed as an alternative to NAFLD.^2^ MAFLD is defined by the presence of hepatic steatosis along with criteria such as diabetes mellitus (DM), overweight/obesity, or metabolic dysfunction.^3,4^ While MAFLD and NAFLD represent similar, although not identical, diseases, approximately 90% of the patient population falls under both definitions.^5^ Therefore, in this review, we have made a deliberate effort to avoid using NAFLD and MAFLD interchangeably and present the data separately. In the definition of MAFLD, the primary clinical measurement has been identified as disease severity, encompassing inflammation and fibrosis stages. However, this has sparked a discussion. The main criticism revolves around the potential to impede awareness of nonalcoholic steatohepatitis, a key endpoint in clinical trials and a crucial factor in drug development.^6,7^ In 2023, Rinella et al recommended the term “metabolic dysfunction-associated steatotic liver disease” (MASLD) after conducting 4 Delphi surveys and 2 face-to-face meetings. The updated terminology defines MASLD as the presence of hepatic steatosis along with at least 1 of the 5 cardiometabolic risk factors. This adjustment ensures the preservation of endpoints from previous clinical trials. Additionally, a new category, MetALD, was introduced to classify patients with MASLD who also exhibit significant alcohol consumption.^8^ Due to the novelty of the terminology and lack of studies investigating the cognitive status of the patients identified as having MASLD, our review was unable to include data on the cognitive status of MASLD patients.

With a reported prevalence of 40%, MAFLD is the most common chronic liver disease globally.^9^ Projected studies indicate that fatty liver prevalence will continue to rise due to the increasing rates of DM and obesity epidemics.^10,11^ Despite primarily manifesting as a liver disease, MAFLD is extensively documented as a multisystemic disorder. Consequently, MAFLD and its associated conditions are under intense scrutiny in industrialized nations. Accumulating evidence highlights the strong association between MAFLD and cardiovascular disorders, DM, metabolic syndrome, obesity, chronic kidney disease, and extrahepatic malignancies, among others.^12^ Recently, MAFLD has emerged as a potential risk factor for the development of cognitive impairment and dementia, attributable to shared risk factors.^13^ In this review, our objective is to provide a comprehensive summary of the current clinical data and elucidate the common pathophysiological mechanisms representing the relationship between these 2 conditions.

## Evidence for Cognitive Impairment in Patients with Fatty Liver Disease

Accumulating evidence indicates a strong association between MAFLD and decreased cognitive function across various domains. Studies based on patient-reported outcomes have shown that patients with NAFLD, without evidence of cirrhosis, experience higher rates of fatigue, memory loss, and concentration problems.^14,15^

One study conducted by Cushman et al^16^ prospectively investigated 30 000 adults aged over 45 years using repetitive tests that assessed word list learning and recall, as well as verbal fluency. The NAFLD was defined based on the fatty liver index, including body mass index, waist circumference, gamma-glutamyl transferase, and triglycerides. The study found that the presence of NAFLD was associated with a two-fold increased risk of cognitive impairment. After adjusting for cardiovascular, stroke, and metabolic risk factors, the risk increased to 2.95-fold.^16^

In another study, middle-aged and elderly patients (n = 1651) were followed up for 4 years and assessed using a mini-mental test adapted according to education level. Nearly half of the population in this study had NAFLD, as defined by ultrasonography. After the 4-year follow-up, 17.7% of patients with NAFLD and 11.7% of patients without NAFLD showed evidence of significantly impaired cognitive performance (*P* < .001).^17^

A systematic review by Georg et al^18^ identified the cognitive domains that were most affected by NAFLD, including general cognition, mental speed, attention and psychomotor speed, as well as ideas, abstraction, figural creations, and mental flexibility.

Not only MAFLD itself, but metabolic disturbances, including DM, have been shown to be associated with cognitive impairment. In a prospective study conducted with 62 obese adults (41 patients with prediabetes and 21 patients with newly diagnosed type 2 DM), the patients’ memory function was evaluated. The contribution of NAFLD and insulin resistance, defined by Homeostasis Model Assessment-Insulin Resistance, to cognitive decline in the memory domain was found to be significant.^19^ Another study using data from the National Health and Nutrition Examination Survey (NHANES) 2011-2014 showed that patients with both NAFLD and type 2 DM performed significantly worse in tasks combining processing speed, sustained attention, and working memory compared to those without type 2 DM.^20^ Additionally, patients with both type 2 DM and NAFLD were reported to have worse fine motor performance. However, it should be noted that they were also less physically active, which is known to be significantly associated with fine motor performance.^21^ Consistent with this evidence, current knowledge indicates that patients with MAFLD experience more severe cognitive impairment compared to those with NAFLD but not MAFLD. In this study, the authors investigated NHANES-III data. Cognitive performance was evaluated using the serial digit learning test (SDLT), the simple reaction time test (SRTT), and the symbol digit substitution test (SDST). The SDLT assessed learning ability, short-term memory, and concentration; the SRTT assessed visual–motor speed and response time; and the SDST assessed processing speed and visual attention. In their analysis, MAFLD patients had significantly lower scores for the SRTT, indicating lower visual–motor speed and response time, after adjusting for sex, age, ethnicity, education level, and history of stroke (odds ratio [OR] = 1.47; 95% CI 1.14-1.89). Moreover, the high likelihood of fibrosis among MAFLD patients was found to be associated with poorer performance on the SRTT and SDST. In fact, the presence of prediabetes among MAFLD patients resulted in a twice worse SRTT performance, while type 2 DM led to a 3-fold worse performance (OR = 2.01; 95% CI 1.37-2.96; OR = 2.87; 95% CI 1.44-5.74, respectively).^13^

Not only have patient-reported studies demonstrated a correlation between decreased cognitive performance and NAFLD, but imaging studies have also supported this finding. In a comparative study, patients with NAFLD exhibited worse results on the Montreal Cognitive Assessment test, which serves as an indicator of cognitive performance, as well as decreased brain tissue volume in magnetic resonance imaging (MRI) compared to those without NAFLD.^22^ Furthermore, in a cross-sectional study conducted with 766 individuals from the Offspring cohort of the Framingham Study, NAFLD was found to be associated with decreased brain volume in MRI after adjusting for age, sex, and metabolic risk factors, suggesting a potential link between NAFLD and brain aging.^23^ In a study by VanWagner et al, it was indeed observed that brain volume was significantly lower among individuals with NAFLD. However, after adjusting for adipose tissue volume and body mass index, this relationship did not remain significant. Lower cerebral perfusion, as a marker of early brain damage, was significantly more common among patients with NAFLD after adjusting for cardiometabolic factors.^24^ Conflicting results have also been reported. In another study conducted among hypertensive and diabetic patients, the presence of hepatic steatosis was not associated with cognitive impairment or brain volume. However, it is worth noting that in this study, the diagnosis of hepatic steatosis was defined using the Dallas steatosis index rather than imaging methods utilized in other studies, which might lead to misclassification of the patients.^25^

The positive correlation between the presence of MAFLD and decreased cognitive performance notwithstanding, there are similar trends in the severity of liver disease and cognitive impairment. It has been shown that MAFLD patients with higher fibrosis scores perform worse in cognitive tests.^13,26^ In a study by Weinstein et al,^26^ however, the authors did not find a significant association between NAFLD and cognitive impairment. Nevertheless, among patients with NAFLD, a higher NAFLD fibrosis score, which indicates a higher probability of advanced fibrosis, was associated with poorer cognitive outcomes, independent of confounding factors.^26^ However, a study that used liver biopsy to diagnose NAFLD found no significant relationship between liver fibrosis stage and the severity of cognitive status. In this study, evidence of hepatic inflammation was linked to poorer cognitive status, as defined by the mini-mental state examination.^27^ In addition to cognitive impairment, depression was found to be a strong predictor of MAFLD due to the effect of chronic inflammation on the gut–brain axis. However, the association of depression with the severity of liver disease was not proven.^28^ Therefore, the current evidence points to a strong association between impaired cognitive status and liver disease itself, as well as its severity. However, there is a need for future research to address this issue.

## Presence of Dementia in Patients with Metabolic Dysfunction-Associated Fatty Liver Disease and Its Relationship to Disease Severity

Despite the existence of shared pathways in the pathophysiological development of MAFLD and dementia, the clinical data regarding the relationship between these 2 conditions remains limited and controversial. Table 1^29-37^ provides a summary of the current studies examining the association between fatty liver disease and dementia. In 2 nationwide Korean studies, a significant correlation was observed between incident dementia and the presence of NAFLD. In the study conducted by Jeong et al, data from 608 994 adults aged ≥60 years, obtained from the Korean National Health Insurance Service database, were analyzed. An elevated fatty liver index, serving as an indicator of NAFLD, was found to be associated with an increased risk of dementia (hazard ratio [HR]: 1.05; 95% CI 1.02-1.08; *P *= .001). Additionally, their study demonstrated a significant inclination toward the development of Alzheimer’s dementia rather than vascular dementia.^29^ Likewise, in a nationwide study conducted with the Korean population, Kim et al^30^ discovered a positive correlation between NAFLD, as determined by the hepatic steatosis index, and dementia. Furthermore, they identified that female and non-obese patients were at higher risk for dementia.^30^ Similarly, in a cross-sectional, population-based study conducted in China, the authors observed a significant association between NAFLD patients with moderate to severe ultrasonographically defined hepatic steatosis and the presence of dementia. These patients had a twice higher risk of all-cause dementia and a 2.65-fold increased risk of vascular dementia compared to patients with mild steatosis. Interleukin 6 was identified as a major factor contributing to the relationship between NAFLD and vascular dementia. However, limitations of this study include the absence of a healthy control group and the evaluation of severity based on steatosis grade, which is a crucial prognostic determinant of NAFLD.^31^ In contrast, among patients with vascular dementia, nearly half of them exhibited ultrasonographically defined NAFLD, with the severity of steatosis grade being associated with more severe neurological profiles.^32^ In a population-based Swedish study, a total of 2898 patients with NAFLD and matched controls, aged 65 years and older, were included from the national database. The study reported a 5-year cumulative incidence rate of 3.6% for dementia in patients with NAFLD, compared to 2% in those without NAFLD. Furthermore, when NAFLD patients had heart disease and stroke as cardiometabolic risk factors, the risk of developing dementia was doubled (HR = 2.00; 95% CI 1.52-2.62).^33^ Currently, there are no studies specifically conducted with the MAFLD population investigating this aspect. However, considering that the cardiometabolic profile of MAFLD patients is known to be more severe compared to NAFLD, it can be speculated that there may be a higher risk for dementia in individuals with MAFLD in the future.^2,38^

Despite these studies, there was no significant association between dementia and NAFLD. A meta-analysis including recent data from 891 562 individuals in 6 countries did find a relationship between NAFLD and cognitive impairment. However, there was no significant association between NAFLD and all-cause dementia (OR = 1.03; 95% CI 0.97-1.09). Interestingly, NAFLD was associated with a decreased risk of vascular dementia compared to Alzheimer’s disease (OR = 0.88; 95% CI 0.79-0.98). It is important to note that the analysis was limited due to a scarcity of studies on this topic.^34^ The results of the Rotterdam study were consistent with these findings. Neither NAFLD nor fibrosis stage increased the incidence of dementia or worsened cognitive status. In fact, a higher fatty liver index was associated with a lower occurrence of dementia (HR = 0.48; 95% CI 0.24-0.94).^35^ It is known that hepatic steatosis and even fibrosis can be reversed with significant weight loss.^37^ Therefore, these findings can be explained by the tendency to lose weight due to dementia.^39^ A previous population-based German study also reported similar results.^36^ However, even though NAFLD itself is not associated with dementia, a more severe fibrosis stage was found to predict dementia, indicating a positive correlation. Further analysis revealed that fibrosis stage increased the hazard ratio for non-Alzheimer dementia rather than Alzheimer’s dementia, suggesting an association between fibrosis stage and a worsened cardiovascular risk profile.^38^

As it is known, all components of metabolic syndrome (hypertension, obesity, insulin resistance, and dyslipidemia) have been linked to systemic inflammation, atherosclerosis, and ultimately, inflammation-induced neurodegeneration.^40^ In light of this knowledge, while the relationship between MAFLD and dementia remains controversial, metabolic syndrome has been proven to be a leading cause of dementia, particularly vascular dementia.^41^ In fact, individual components of metabolic syndrome have also been shown to increase the risk of dementia. Therefore, it is recommended to evaluate patients for cognitive impairment if 1 or 2 components of metabolic syndrome are present in their medical history.^42^ The Westernized diet has been proposed to cause systemic inflammation and alterations in the gut–brain axis.^43^ For this reason, the Mediterranean diet has been suggested as a helpful approach for the prevention of both MAFLD and cognitive decline, as diet and lifestyle are considered modifiable factors for the prevention of both MAFLD and dementia.^43,44^

## Mechanisms Linking Metabolic (Dysfunction) Associated Fatty Liver Disease and Cognitive Impairment

The accumulating evidence supports the relationship between MAFLD and cognitive impairment. Although the relationship between MAFLD and dementia remains controversial, there are common pathways that contribute to the development of both diseases over a prolonged period of time.^35^ These common pathways include systemic inflammation, insulin resistance, vascular dysfunction, gut dysbiosis, and hyperammonemia.^45^

Systemic inflammation, starting from the liver and causing neuroinflammation, triggers the development of Alzheimer’s dementia due to shared inflammatory pathways. In a mouse model, decreased expression of low-density lipoprotein receptor-related protein 1, which plays a significant role in β-amyloid clearance, has been observed.^46^ In another diet-induced mouse model, changes in lipid homeostasis resulted in increased expression of Tnfα, Cox2, p21, and Nox2, leading to increased oxidative stress and brain inflammation. Mice fed a NAFLD diet showed decreased cerebral perfusion and increased cognitive impairment.^47^ Moreover, aging itself is proposed to result in alterations in the balance of pro-inflammatory and anti-inflammatory cytokines, which is described as the inflamm-aging theory.^48^ Finally, chronic inflammation triggered by the activation of the nuclear factor-kappa B pathway and continuous secretion of pro-inflammatory cytokines contribute to the development of NAFLD and cognitive impairment.^49^

Insulin signaling pathways, such as the IGF-1/IGF-1 receptor-induced phosphoinositide 3-kinase/Akt signaling pathway, are involved in axon development and synaptic formation, ultimately contributing to neuronal plasticity and memory formation. Therefore, insulin resistance triggers neuronal damage and cognitive impairment.^50^ Furthermore, the association between insulin resistance and vascular injury is well established. Vascular injury and atherosclerosis have been speculated to be key factors in the development of vascular dementia.^23^ Indeed, dementia is more prevalent among patients with type 2 diabetes mellitus. Therefore, the regulation of blood glucose levels and the management of diabetes could be considered common therapeutic targets for both MAFLD and dementia.^51^ Similarly, hypertension and atherosclerosis are other shared targets in the management of MAFLD and dementia. These vascular complications have been shown to alter total cerebral blood flow and microcirculation, leading to cognitive impairment in patients with NAFLD.^24^

The gut microbiota has been identified as playing a crucial role in the communication between the gut and the brain. Changes in the microbiota can lead to an increase in insulin resistance and intestinal permeability, ultimately resulting in chronic inflammation.^52^ In fact, the use of probiotics is recommended as a therapeutic option in the management of MAFLD and even for patients with psychiatric disorders such as depression.^52^ Furthermore, certain products of bacterial metabolism, such as endotoxins and ammonia, can induce neuroinflammation. Hyperammonemia, caused by dysbiosis and decreased liver metabolism, makes the blood–brain barrier more susceptible and leads to hepatic encephalopathy and cognitive impairment.^53^

## Conclusion

Both MAFLD and dementia are currently recognized as significant issues in the industrialized world, primarily due to changes in diet and an aging population. While there are conflicting results, accumulating evidence suggests that MAFLD is associated with cognitive impairment, likely through shared pathological pathways. Timely detection and management of metabolic comorbidities might be beneficial in the prevention of dementia.

## Figures and Tables

**Table 1.** Summary of Articles Investigating the Relationship Between Fatty Liver Disease and Dementia

Author	Year	Diagnosis of Hepatic Steatosis	Type of Dementia	Diagnosis of Dementia	Population	Follow-up Time	Result
Jeong et al^29^	2022	Fatty liver index	Vascular dementia and Alzheimer’s disease	ICD-10 codes or presence of dementia associated medication use	608 994 adults aged ≥60 years from Korean National Health Insurance Service database	11 years	Higher fatty liver disease is associated with higher risk of incident dementia.
Kim et al^30^	2022	Hepatic steatosis index	NA	ICD-10 codes	4 031 948 adults aged 40-69 years from Korean National Health Insurance Service database	9.5 years	NAFLD patients defined by hepatic steatosis index are under higher risk for dementia development.
Wang et al^31^	2022	Ultrasonography	All-cause dementia	ICD-10 codes	5129 Chinese participants from rural regions aged ≥60 years	NA	Moderate to severe NAFLD was significantly associated with dementia, particularly with vascular dementia.
Moretti et al^32^	2022	Ultrasonography	Vascular dementia	CT/MRI	285 patients from a tertiary care center	NA	Patients with NAFLD and vascular dementia had worse neuropsychological outcomes and a worse metabolic profile.
Shang et al^33^	2022	ICD-10 codes	All-cause dementia	ICD-10 codes	2898 patients with NAFLD and 28 357 matched controls from Swedish cause of death register 2016	5.5 years	NAFLD and coexistence of cardiometabolic risk factors increased the risk for dementia.
Wang et al^34^	2022	Noninvasive tests, liver biopsy and ICD-10 codes	All-cause dementia and cognitive impairment	ICD-10 codes	Meta-analysis 891 562 individuals from 6 countries	3-9.5 years	NAFLD is associated with increased risk for cognitive impairment but not with all-cause dementia.
Xiao et al^35^	2022	Fatty liver index, ultrasonography, Fibroscan	All-cause dementia and cognitive impairment	*Diagnostic and Statistical Manual of Mental Disorders*, Third Edition	3975 participants from Rotterdam study	15.5 years	NAFLD is a protective factor against development of dementia.
Labenz et al^36^	2021	ICD-10 codes	All-cause dementia	ICD-10 codes	A population-based German study consisted of 22 317 patients with NAFLD were matched to 22 317 patients without NAFLD	10 years	NAFLD and development of dementia are not associated.
Shang et al^37^	2020	Liver biopsy	All-cause dementia	ICD-10	656 NAFLD patients underwent liver biopsy and 6436 matched controls	19.7 years	NAFLD and dementia are not associated. However, increased fibrosis stage may be predictor of dementia.

CT, computed tomography; MRI, magnetic resonance imaging; NA, not applicable; NAFLD, nonalcoholic fatty liver disease.

## References

[1] Younossi ZM Golabi P Paik JM Henry A Van Dongen C Henry L . The global epidemiology of nonalcoholic fatty liver disease (NAFLD) and nonalcoholic steatohepatitis (NASH): a systematic review. Hepatology. 2023;77(4):1335 1347. (10.1097/HEP.0000000000000004) 36626630 PMC10026948

[2] Kaya E Yilmaz Y . Epidemiology, natural history, and diagnosis of metabolic dysfunction-associated fatty liver disease: a comparative review with nonalcoholic fatty liver disease. Ther Adv Endocrinol Metab. 2022;13:20420188221139650. (10.1177/20420188221139650) 36533185 PMC9747887

[3] Eslam M Newsome PN Sarin SK , et al. A new definition for metabolic dysfunction-associated fatty liver disease: an international expert consensus statement. J Hepatol. 2020;73(1):202 209. (10.1016/j.jhep.2020.03.039) 32278004

[4] Méndez-Sánchez N Bugianesi E Gish RG , et al. Global multi-stakeholder endorsement of the MAFLD definition. Lancet Gastroenterol Hepatol. 2022;7(5):388 390. (10.1016/S2468-1253(22)00062-0) 35248211

[5] Wong VW Wong GL Woo J , et al. Impact of the new definition of metabolic associated fatty liver disease on the epidemiology of the disease. Clin Gastroenterol Hepatol. 2021;19(10):2161 2171.e5. (10.1016/j.cgh.2020.10.046) 33137486

[6] Yilmaz Y . The heated debate over NAFLD renaming: an ongoing saga. Hepatol Forum. 2023;4(3):89 91. (10.14744/hf.2023.2023.0044) 37822312 PMC10564248

[7] Yilmaz Y Zeybel M Adali G , et al. TASL practice guidance on the clinical assessment and management of patients with nonalcoholic fatty liver disease. Hepatol Forum. 2023;4(suppl 1):1 32. (10.14744/hf.2023.2023.0011) PMC10588738 37920782

[8] Rinella ME Lazarus JV Ratziu V , et al. A multisociety Delphi consensus statement on new fatty liver disease nomenclature. J Hepatol. 2023;79(6):1542 1556. (S0168-8278(23)00418-X) 37364790 10.1016/j.jhep.2023.06.003

[9] Lim GEH Tang A Ng CH , et al. An observational data meta-analysis on the differences in prevalence and risk factors between MAFLD vs NAFLD. Clin Gastroenterol Hepatol. 2023;21(3):619 629.e7. (10.1016/j.cgh.2021.11.038) 34871813

[10] Estes C Anstee QM Arias-Loste MT , et al. Modeling NAFLD disease burden in China, France, Germany, Italy, Japan, Spain, United Kingdom, and United States for the period 2016-2030. J Hepatol. 2018;69(4):896 904. (10.1016/j.jhep.2018.05.036) 29886156

[11] Estes C Chan HLY Chien RN , et al. Modelling NAFLD disease burden in four Asian regions-2019-2030. Aliment Pharmacol Ther. 2020;51(8):801 811. (10.1111/apt.15673) 32133676 PMC7154715

[12] Kaya E Yilmaz Y . Metabolic-associated fatty liver disease (MAFLD): a multi-systemic disease beyond the liver. J Clin Transl Hepatol. 2022;10(2):329 338. (10.14218/JCTH.2021.00178) 35528971 PMC9039705

[13] Yu Q He R Jiang H , et al. Association between metabolic dysfunction-associated fatty liver disease and cognitive impairment. J Clin Transl Hepatol. 2022;10(6):1034 1041. (10.14218/JCTH.2021.00490) 36381086 PMC9634777

[14] Doward LC Balp MM Twiss J , et al. Development of a patient-reported outcome measure for non-alcoholic steatohepatitis (NASH-CHECK): results of a qualitative study. Patient. 2021;14(5):533 543. (10.1007/s40271-020-00485-w) 33336323 PMC8357766

[15] Elliott C Frith J Day CP Jones DE Newton JL . Functional impairment in alcoholic liver disease and non-alcoholic fatty liver disease is significant and persists over 3 years of follow-up. Dig Dis Sci. 2013;58(8):2383 2391. (10.1007/s10620-013-2657-2) 23609794

[16] Cushman M Callas PW Alexander KS , et al. Nonalcoholic fatty liver disease and cognitive impairment: a prospective cohort study. PLoS One. 2023;18(4):e0282633. (10.1371/journal.pone.0282633) 37058527 PMC10104321

[17] Liu Q Liu C Hu F Deng X Zhang Y . Non-alcoholic fatty liver disease and longitudinal cognitive changes in middle-aged and elderly adults. Front Med (Lausanne). 2021;8:738835. (10.3389/fmed.2021.738835) 35111769 PMC8803120

[18] George ES Sood S Daly RM Tan SY . Is there an association between non-alcoholic fatty liver disease and cognitive function? a systematic review. BMC Geriatr. 2022;22(1):47. (10.1186/s12877-021-02721-w) 35016619 PMC8753832

[19] Vadini F Simeone PG Desideri G , et al. Insulin resistance and NAFLD may influence memory performance in obese patients with prediabetes or newly-diagnosed type 2 diabetes. Nutr Metab Cardiovasc Dis. 2021;31(9):2685 2692. (10.1016/j.numecd.2021.05.027) 34226120

[20] Weinstein AA de Avila L Paik J , et al. Cognitive Performance in Individuals with Non-Alcoholic Fatty Liver Disease and/or type 2 diabetes mellitus. Psychosomatics. 2018;59(6):567 574. (10.1016/j.psym.2018.06.001) 30086995

[21] Weinstein AA Ngo D de Avila L , et al. Association of physical activity and fine motor performance in individuals with type 2 diabetes mellitus and/or non-alcoholic fatty liver disease. Ann Med. 2023;55(1):1345 1353. (10.1080/07853890.2023.2193422) 36974658 PMC10054279

[22] Filipović B Marković O Đurić V Filipović B . Cognitive changes and brain volume reduction in patients with nonalcoholic fatty liver disease. Can J Gastroenterol Hepatol. 2018;2018:9638797. (10.1155/2018/9638797) 29682494 PMC5848059

[23] Weinstein G Zelber-Sagi S Preis SR , et al. Association of nonalcoholic fatty liver disease with lower brain volume in healthy middle-aged adults in the Framingham study. JAMA Neurol. 2018;75(1):97 104. (10.1001/jamaneurol.2017.3229) 29159396 PMC5833484

[24] VanWagner LB Terry JG Chow LS , et al. Nonalcoholic fatty liver disease and measures of early brain health in middle-aged adults: the CARDIA study. Obesity (Silver Spring). 2017;25(3):642 651. (10.1002/oby.21767) 28169509 PMC5323279

[25] Basu E Mehta M Zhang C , et al. Association of chronic liver disease with cognition and brain volumes in two randomized controlled trial populations. J Neurol Sci. 2022;434:120117. (10.1016/j.jns.2021.120117) 34959080 PMC8957528

[26] Weinstein G Davis-Plourde K Himali JJ Zelber-Sagi S Beiser AS Seshadri S . Non-alcoholic fatty liver disease, liver fibrosis score and cognitive function in middle-aged adults: the Framingham Study. Liver Int. 2019;39(9):1713 1721. (10.1111/liv.14161) 31155826 PMC6736704

[27] Tuttolomondo A Petta S Casuccio A , et al. Reactive hyperemia index (RHI) and cognitive performance indexes are associated with histologic markers of liver disease in subjects with non-alcoholic fatty liver disease (NAFLD): a case control study. Cardiovasc Diabetol. 2018;17(1):28. (10.1186/s12933-018-0670-7) 29452601 PMC5815178

[28] Yang S Cheng J Zhang R , et al. Metabolic dysfunction-associated fatty liver disease and liver fibrosis: prevalence and associated factors in the middle-aged and older US population. Hepatol Res. 2022;52(2):176 186. (10.1111/hepr.13728) 34751487

[29] Jeong S Oh YH Choi S , et al. Association of non-alcoholic fatty liver disease with incident dementia later in life among elder adults. Clin Mol Hepatol. 2022;28(3):510 521. (10.3350/cmh.2021.0332) 35299291 PMC9293607

[30] Kim GA Oh CH Kim JW , et al. Association between non-alcoholic fatty liver disease and the risk of dementia: a nationwide cohort study. Liver Int. 2022;42(5):1027 1036. (10.1111/liv.15244) 35289469

[31] Wang Y Li Y Liu K , et al. Nonalcoholic fatty liver disease, serum cytokines, and dementia among rural-dwelling older adults in China: a population-based study. Eur J Neurol. 2022;29(9):2612 2621. (10.1111/ene.15416) 35608965

[32] Moretti R Giuffré M Crocè LS Gazzin S Tiribelli C . Nonalcoholic fatty liver disease and altered neuropsychological functions in patients with subcortical vascular dementia. J Pers Med. 2022;12(7):1106. (10.3390/jpm12071106) 35887603 PMC9323787

[33] Shang Y Widman L Hagström H . Nonalcoholic fatty liver disease and risk of dementia: a population-based cohort study. Neurology. 2022;99(6):e574 e582. (10.1212/WNL.0000000000200853) 35831178 PMC9442617

[34] Wang L Sang B Zheng Z . Risk of dementia or cognitive impairment in non-alcoholic fatty liver disease: a systematic review and meta-analysis. Front Aging Neurosci. 2022;14:985109. (10.3389/fnagi.2022.985109) 36204558 PMC9530447

[35] Xiao T van Kleef LA Ikram MK de Knegt RJ Ikram MA . Association of nonalcoholic fatty liver disease and fibrosis with incident dementia and cognition: the Rotterdam study. Neurology. 2022;99(6):e565 e573. (10.1212/WNL.0000000000200770) 35618435 PMC9442616

[36] Labenz C Kostev K Kaps L Galle PR Schattenberg JM . Incident dementia in elderly patients with nonalcoholic fatty liver disease in Germany. Dig Dis Sci. 2021;66(9):3179 3185. (10.1007/s10620-020-06644-1) 33037968 PMC8379104

[37] Shang Y Nasr P Ekstedt M , et al. Non-alcoholic fatty liver disease does not increase dementia risk although histology data might improve risk prediction. JHEP Rep. 2021;3(2):100218. (10.1016/j.jhepr.2020.100218) 33554097 PMC7847958

[38] Kaya E Yilmaz Y . Insidious danger for young adults: metabolic (dysfunction)-associated fatty liver disease. Hepatol Forum. 2022;3(2):39 40. (10.14744/hf.2022.2022.0002) 35783479 PMC9243761

[39] Hamurcu Varol P Kaya E Alphan E Yilmaz Y . Role of intensive dietary and lifestyle interventions in the treatment of lean nonalcoholic fatty liver disease patients. Eur J Gastroenterol Hepatol. 2020;32(10):1352 1357. (10.1097/MEG.0000000000001656) 32092046

[40] Borshchev YY Uspensky YP Galagudza MM . Pathogenetic pathways of cognitive dysfunction and dementia in metabolic syndrome. Life Sci. 2019;237:116932. (10.1016/j.lfs.2019.116932) 31606384

[41] Tahmi M Palta P Luchsinger JA . Metabolic syndrome and cognitive function. Curr Cardiol Rep. 2021;23(12):180. (10.1007/s11886-021-01615-y) 34668083

[42] Machado-Fragua MD Fayosse A Yerramalla MS , et al. Association of metabolic syndrome with incident dementia: role of number and age at measurement of components in a 28-year follow-up of the Whitehall II cohort study. Diabetes Care. 2022;45(9):2127 2135. (10.2337/dc22-0206) 35819815 PMC9472484

[43] Więckowska-Gacek A Mietelska-Porowska A Wydrych M Wojda U . Western diet as a trigger of Alzheimer’s disease: from metabolic syndrome and systemic inflammation to neuroinflammation and neurodegeneration. Ageing Res Rev. 2021;70:101397. (10.1016/j.arr.2021.101397) 34214643

[44] Eslam M Sarin SK Wong VW , et al. The Asian Pacific Association for the Study of the Liver clinical practice guidelines for the diagnosis and management of metabolic associated fatty liver disease. Hepatol Int. 2020;14(6):889 919. (10.1007/s12072-020-10094-2) 33006093

[45] Cheon SY Song J . Novel insights into non-alcoholic fatty liver disease and dementia: insulin resistance, hyperammonemia, gut dysbiosis, vascular impairment, and inflammation. Cell Biosci. 2022;12(1):99. (10.1186/s13578-022-00836-0) 35765060 PMC9237975

[46] Pinçon A De Montgolfier O Akkoyunlu N , et al. Non-alcoholic fatty liver disease, and the underlying altered fatty acid metabolism, reveals brain hypoperfusion and contributes to the cognitive decline in APP/PS1 mice. Metabolites. 2019;9(5):104. (10.3390/metabo9050104) 31130652 PMC6572466

[47] Kim DG Krenz A Toussaint LE , et al. Non-alcoholic fatty liver disease induces signs of Alzheimer’s disease (AD) in wild-type mice and accelerates pathological signs of AD in an AD model. J Neuroinflammation. 2016;13:1. (10.1186/s12974-015-0467-5) 26728181 PMC4700622

[48] Ju Hwang C Choi DY Park MH Hong JT . NF-κB as a key mediator of brain inflammation in Alzheimer’s disease. CNS Neurol Disord Drug Targets. 2019;18(1):3 10. (10.2174/1871527316666170807130011) 28782486

[49] Kucukoglu O Sowa JP Mazzolini GD Syn WK Canbay A . Hepatokines and adipokines in NASH-related hepatocellular carcinoma. J Hepatol. 2021;74(2):442 457. (10.1016/j.jhep.2020.10.030) 33161047

[50] Dyer AH Vahdatpour C Sanfeliu A Tropea D . The role of insulin-like growth factor 1 (IGF-1) in brain development, maturation and neuroplasticity. Neuroscience. 2016;325:89 99. (10.1016/j.neuroscience.2016.03.056) 27038749

[51] Dichgans M Zietemann V . Prevention of vascular cognitive impairment. Stroke. 2012;43(11):3137 3146. (10.1161/STROKEAHA.112.651778) 22935401

[52] Socała K Doboszewska U Szopa A , et al. The role of microbiota-gut-brain axis in neuropsychiatric and neurological disorders. Pharmacol Res. 2021;172:105840. (10.1016/j.phrs.2021.105840) 34450312

[53] Kjærgaard K Mikkelsen ACD Wernberg CW , et al. Cognitive Dysfunction in Non-Alcoholic Fatty Liver Disease-Current Knowledge, Mechanisms and Perspectives. J Clin Med. 2021;10(4):673. (10.3390/jcm10040673) 33572481 PMC7916374

